# Nanomedicines and Nanosimilars—Why a Robust Centralised Regulatory Framework Is Essential to Enhance Patient Safety

**DOI:** 10.3389/fphar.2021.787239

**Published:** 2022-02-24

**Authors:** Michael P. Isles

**Affiliations:** European Alliance for Access to Safe Medicines, Dublin, Ireland

**Keywords:** advocacy program, centralised regulatory procedure, hybrid application, nanomedicines, nanosimilars, follow-on products

Given that nanomedicines and follow-on nanosimilars have complex manufacturing processes and heteromolecular structures, the question is being raised in ever increasing frequency as to whether the current European regulation of medicines for human use is robust enough to authorise these medicinal products and their follow-ons. Until this can be achieved, there is a potential for patient safety to be compromised.

The current situation is that nanomedicines have the potential for being assessed under four different types of procedures: the national procedure, the decentralised procedure, the mutual recognition procedure, and the centralised procedure. In this context, it is important to note that a survey published in 2018 reported “…strong regional differences in the regulation of nanomedicines and confirmed the need for a harmonisation of information requirements on nano-specific properties” (Bremer-Hoffmann et al., 2018). Given their complex nature and the fact that each nanomedicine will have unique features, there is currently a lack of guidelines or protocols so that these medicines can be appropriately processed, which will provide a marketing authorisation (MA) that meets the demanding standards of today and thus ensure patient safety (Nanomedicines and Nanosimilars, 2021).

The EU Nanomedicines Regulatory Coalition (Nanomedicines Regulatory Coalition, 2021) currently comprising seven pan-European organisations is therefore advocating for all nanomedicines to be assessed by the EMA Centralised regulatory procedure (Patient Safety and Nanomedicines, 2020).

This is equally true of the off-patent follow-on copy products, or nanosimilars, as they are also called. Within this context, a centralised regulatory process that addresses this is needed at the EU level, and in the absence of a tailored regulatory pathway similar to that of the biosimilars, the European Alliance for Access to Safe Medicines (EAASM) strongly believes that all future nanosimilars should go through the Hybrid Application process (10.3) and not the Generics Application process (10.1). This pathway, if consistently applied and aligned to the draft guidance (European Medicines Agency, 2015) which the EMA has produced for specific types of nanomedicines, would ensure that follow-on copies are therapeutically similar to their originator and therefore improve patient safety.

There will be different manufacturers producing these similar products from different sites with differing manufacturing processes, and so the production of identical replicas of the originator product cannot ever be achieved (Ehmann et al., 2013; Marden et al., 2018). It is for this reason that a thorough clinical valuation must be carried out before an MA can be granted.

Patient harm has occurred when a nanosimilar has not had this rigorous safety and efficacy check established through a clinical trial (Rottembourg et al., 2011a) program.

This article endeavors to lay out the critical success factors that will enable a centralised procedure for nanomedicines and nanosimilars to be achieved.

## Methodology

The recommendations of this article have been developed due to extensive desk research (Patient Safety and Nanomedicines, 2020) and in consultation with field experts in one-on-one interviews and through two round-tables which took place in the European Parliament in April 2019 (Event summary, 2019) and November 2020 and which were fully reported.

This has enabled the EAASM to adopt a robust strategy of a continuous extensive advocacy program with all influential stakeholders and the EU Institutions (Nanomedicines Regulatory Coalition, 2021).

This strategy aims to raise the political temperature (Letter to Commissioner for Health and Food Safety Ms. Stella Kyriakides, 2021) so that even more focus can be placed on the regulatory institutions to ensure that a fit-for-purpose regulatory pan-European procedure is adopted as quickly as possible.

**FIGURE 1:**
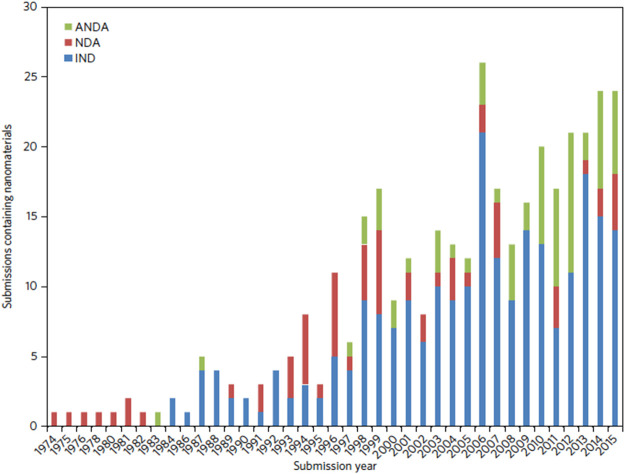
Number of nanomaterial product applications submitted to CDER by year. Applications are separated as INDs, NDAs, and ANDAs.

The need for a harmonised centralised regulatory procedure is highlighted by three key factors:

1) The plethora of nanomedicines in the pipeline (see Figure 1; Table 1; Van Trier, 2021) which indicates the diversity and complexity of these medicines
2) The evolution of many NBCD marketing authorisations (of which many are nanomedicines and nanosimilars—see Table 2) adapted by Klein et al. (2019) which show the diverse nature of the regulatory routes. This gives rise to different national health agencies assessing these medicines and allows for the marketing of different brand names, which in turn makes PV linkage difficult and thus compromises patient safety.
3) Interchangeability of “similar” medicines requires strong central guidelines and education programs to ensure that hospital- and community-based policies are implemented by doctors, pharmacists, and nurses in a coordinated way.

**TABLE 1:** Overview of the commercially available nanomedicines in the EU (Van Trier, 2021).

Nanomedicine class	Active substance	Brand name	Pharmaceutical form	Indication
Nanoparticles	Albumine-particle	Abraxane	Powder for suspension	Breast neoplasm
	Bound paclitaxel			Non–small-cell lung cancer
				Pancreatic neoplasms
	Y90 ibritumab tiuxetan	Zevalin	Solution for infusiion	Folicullar lymphoma
	Glatirimer acetate	Copaxone	Solution for injection	Multiple sclerosis
Liposome	Cytarabine	DepoCyt	Suspension for injection	Lymphomatous meningitis
	Mifamurtide	*Mepact	Powder for concentrate for dispersion for infusion	Osteosarcoma
	Morphine	DepoDur	Suspension for injection	Pain
	Doxorubicin hydrochloride	Caelyx	Concentrate for suspension for infusion	Kaposi sarcoma
	Doxorubicin hydrochloride	Myocet	Powder, dispersion, and solvent for concentrate for infusion	Metastatic breast cancer
	Amphotericine B	AmBisome	Powder for solution for infusion	Fungal infection
	Daunorubicin	DaunoXome	Concentrate for solution for infusion	HIV-related Kaposi sarcoma
	Cytaribine daunorubicin	*Vyxeos	Concentrate for solution for infusion	Leukemia
	Amikacinesulfaat	*Arikayce lyposomal	Nebulizer dispersion	NTM lung diseases
	Irinotecan	*Onyvide	Solution for infusion	Pancreatic cancer
Lipid nanoparticles	mRNA encoding for SARS-CoV-2 Spike protein	Comirnaty	Concentrate for dispersion for injection	COVID-19
	Patisian	Onpattro	Intravenous infusion	Polyneuropathy of hereditary TTR-mediated amyloidosis (hATTR)
	ChAdOx1-S	Vaxzevria	Suspension for injection	COVID-19
	Encoding the			
	SARSCoV-2			
	Spike glycoprotein			
	mRNA encoding	Spikevax	Dispersion for injection	COVID-19
	for SARS-CoV-2			
	Spike protein			
Nanocrystals	Paliperidone palmitate	Xeplion	Prolonged release suspension for injection	Schizophrenia
	Onlazapine pamoate	Zypadhera	Powder and solvent for prolonged release suspension for injection	Schizophrenia
	Aprepitant	Emend	Capsule	Nausea and vomiting
	Fenofibrate	Tricor	Tablet	Hyperlipidaemia
		Lipanthyl		
		Lipidil		
Iron-carbohydrates	Feric carboxymaltose	Ferinject	Solution for infusion	Iron deficiency
	Iron (3) isomaltoside	Monofer	Solution for infusion	Iron deficiency
	Iron (3)-hydroxide	Ferrosat	Solution for infusion	Iron deficiency
	Dextran complec			

*Designated orphan medicine

**TABLE 2:** Overview of NBCD follow-on products approved in the EU *via* the three abbreviated applications: generic, hybrid, and biosimilar pathways, as well as new applications by originator companies *via* informed consent, sorted by authorization date since the first approval in 1999 until November 2018.

Reference product (MAH)	Follow-on product (MAH)^a^	Authorisation date	Authorisation procedure	RMS (if applicable)	Application procedure
Venofer^®^ 20 mg/ml (Vifor) Iron sucrose complex					
	Ferrovin (Refarm)	27-01-2005	NP (GR, MT)	N/A	Article 10 (1)
	Óxido Férrico Sacarosado Generis (Generis Farmacêutica)	28-05-2007	NP (PT)	N/A	Article 10 (1)
	Hemafer-S (Uni-Pharma)	16-07-2008	NP (GR)	N/A	Article 10 (1)
	Faremio (Demo)	26-08-2008	NP (GR)	N/A	Article 10 (1)
	Dextrifer-S (Intermed)	28-08-2008	NP (GR)	N/A	Article 10 (1)
	Intrafer (Vianex)	01-09-2008	NP (GR)	N/A	Article 10 (1)
	Fer Sandoz (Sandoz)	05-09-2008	NP (FR)	N/A	Article 10 (1)
	Óxido Férrico Sacarosado Accord (Accord Healthcare)	09-10-2008	NP (PT)	N/A	Article 10 (1)
	Fer Mylan (Mylan)	27-10-2008	NP (FR)	N/A	Article 10 (1)^b^
	Alvofer (Cooper Pharmaceuticals)	13-11-2008	NP (GR)	N/A	Article 10 (1)
	Ferrinemia (Help Pharmaceuticals)	21-11-2008	NP (GR, MT)	N/A	Article 10 (1)
	Ironcrose (Target Pharma)	21-11-2008	NP (GR)	N/A	Article 10 (1)
	Venotrix (Alternova)	12-02-2009	NP (FI)	N/A	Article 10 (1)
	IJzerhydroxide sacharose complex (Teva)	18-02-2009	NP (NL)	N/A	Article 10 (1)
	Nefro-Fer (Medice Arzneimittel Pütter)	15-03-2009	DCP	DE	Article 10 (1)
	Veniron (Viofar)	17-06-2010	NP (GR)	N/A	Article 10 (1)
	Nephroferol (Verisfield)	10-01-2011	NP (GR)	N/A	Article 10 (1)
	Reoxyl (Medicus)	04-01-2012	NP (GR)	N/A	Article 10 (1)
	Järnsackaros Rechon (Rechon Life Science)	14-03-2012	NP (SE)	N/A	Article 10 (1)
	Ferracin (Acino)	26-07-2012	NP (NL)	N/A	Article 10 (1)
	Fer Panpharma (Panmedica)	10-02-2014	NP (FR)	N/A	Article 10 (1)
	Sucrofer (Claris Lifesciences)	01-06-2018	DCP	United Kingdom	Article 10 (3)
Copaxone^®^ 20 mg/ml (Teva) Glatiramer acetate					
	Brabio (Synthon)	10-05-2016	DCP	NL	Article 10 (3)
	Sclerthon (Synthon)	10-05-2016	DCP	NL	Article 10 (3)
	Glatiramer acetate Mylan (Mylan)	10-05-2016	DCP	NL	Article 10 (3)
	Glatiramer acetate Teva (Teva)	18-09-2018	DCP	DE	Article 10(c)
	Copaxone^®^ 40 mg/ml (Teva) Glatiramer acetate				
	Glatiramer acetate Alvogen (Alvogen)	02-11-2017	DCP	NL	Article 10 (3)
	Glatiramer acetate Mylan (Mylan)	02-11-2017	DCP	NL	Article 10 (3)
	Marcyto (Synthon)	02-11-2017	DCP	NL	Article 10 (3)
	Sclerthon (Synthon)	02-11-2017	DCP	NL	Article 10 (3)
	Glatiramer acetate Teva (Teva)	18-09-2018	DCP	DE	Article 10(c)
Renvela^®^ 800 mg (Genzyme)					
	Sevelamer carbonate Sevelamer carbonate AL (Aliud Pharma)	12-03-2014	DCP	DK	Article 10 (3)
	Sevelamer carbonate Teva (Teva)	23-04-2014	DCP	DK	Article 10 (3)
	Sevelamer carbonate Synthon (Synthon)	22-05-2014	DCP	DK	Article 10 (3)
	Sevelamer carbonate Housthon (Amneal Pharma Europe)	22-05-2014	DCP	DK	Article 10 (3)
	Sevelamer carbonate Aurobindo (Aurobindo Pharma)	22-05-2014	DCP	DK	Article 10 (3)
	Sevelamer carbonate Sandoz (Sandoz)	22-05-2014	DCP	DK	Article 10 (3)
	Sevelamer carbonate Genthon (Genthon)	22-05-2014	DCP	DK	Article 10 (3)
	Sevelamer carbonate Mylan (Mylan)	22-05-2014	DCP	DK	Article 10 (3)
	Sevelamer carbonate Sandoz (Sandoz)	22-05-2014	DCP	DK	Article 10 (3)
	Sevelamer carbonate Heaton (Heaton)	22-05-2014	DCP	CZ	Article 10 (3)
	Sevemed (Medice Arzneimittel Pütter)	18-06-2014	DCP	DK	Article 10 (3)
	Sevelamer carbonate Stada (Centrafarm B.V.)	18-08-2014	DCP	DK	Article 10 (3)
	Sevelamer carbonate Zentiva (Genzyme)	14-01-2015	CP	N/A	Article 10(c)
	Sevelamer carbonate Ratiopharm (Ratiopharm)	16-03-2015	DCP	DK	Article 10 (3)
	Sevelamer carbonate Arrow (Arrow Generiques)	16-11-2017	NP (FR)	N/A	Article 10 (3)b
Renvela^®^ 2.4 g (Genzyme)					
	Sevelamer carbonate Sevelamer carbonate Zentiva (Genzyme)	14-01-2015	CP	N/A	Article 10(c)
	Sevelamer carbonate Sandoz (Sandoz)	15-09-2015	DCP	DK	Article 10 (3)
	Sevelamer carbonate Genthon (Genthon)	30-09-2016	DCP	DK	Article 10 (3)
	Fosquel (Avansor Pharma)	30-09-2016	DCP	DK	Article 10 (3)
	Sevelamer carbonate Stada (Stada Arzneimittel)	17-10-2016	DCP	DK	Article 10 (3)
	Sevelamer carbonate Aurobindo (Aurobindo Pharma B.V.)	16-02-2017	NP (NL)	N/A	Article 10 (3)
	Sevemed (Medice Arzneimittel Pütter)	05-04-2017	DCP	DK	Article 10 (3)
	Sevelamer carbonate Mylan (Mylan)	08-05-2017	DCP	DK	Article 10 (3)
	Sevelamer carbonate Arrow (Arrow Generiques)	13-06-2017	NP (FR)	N/A	Article 10 (3)b
	Sevelamer carbonate Aurobindo (Aurobindo Pharma)	05-07-2017	DCP	DK	Article 10 (3)
Diprivan^®^ 10 mg/ml (Aspen) Propofol					
	Propofol (Genthon)	10-08-1999	MRP	United Kingdom	Article 10 (1)
	Propofol Lipuro (B. Braun)	11-12-1999	MRP/NP	DE	Article 10 (1)
	Propofol Genthon (Genthon)	06-03-2000	NP (NL)	N/A	Article 10 (1)
	Propofol MCT/LCT Fresenius (Fresenius Kabi)	18-01-2005	MRP	DE	Article 10 (1)
	Propofol Claris (Claris Lifesciences)	27-03-2006	MRP	NL	Article 10 (1)
	Propofol Panpharma (Claris Lifesciences)	18-06-2008	NP (FR)	N/A	Article 10 (1)
	Propofol Lipuroc ^c^(B. Braun)	14-07-2008	DCP	DE	Article 10 (3)
	Propofol Primexd (Primex Pharmaceuticals)	17-04-2009	MRP	FI	Article 10 (1)
	Propofol Norameda (UAB Norameda)	28-04-2011	DCP	DE	Article 10 (1)
	Propofol BioQ Pharma (BioQ Pharma)	06-07-2012	DCP	NL	Article 10 (1)
	Propofol Sandoz (Sandoz)	06-07-2012	DCP	NL	Article 10 (1)
	Ripol (Corden Pharma)	21-02-2013	DCP	IT	Article 10 (1)
	Propofol MCT/LCT Fresenius pre-filled syringe (Fresenius Kabi)	12-03-2013	DCP	DE	Article 10 (1)
	Propofol Demo (Demo)	03-05-2017	DCP	PT	Article 10 (1)
Diprivan^®^ 20 mg/ml (Aspen) Propofol					
	Propofol Genthon (Genthon)	06-03-2000	NP (NL)	N/A	Article 10 (1)
	Propofol (Genthon)	08-08-2000	MRP	United Kingdom	Article 10 (1)
	Propofol 2% (Fresenius Kabi)	21-05-2001	MRP/NP	DE	Article 10 (1)
	Propofol Lipuroc ^c^(B. Braun)	02-12-2001	MRP/NP	DE	Article 10 (1)
	Propofol Mylan (Mylan)	05-05-2003	NP (FR)	N/A	Article 10 (1)
	Propofol MCT/LCT Fresenius (Fresenius Kabi)	18-01-2005	MRP	DE	Article 10 (1)
	Propofol Claris (Claris Lifesciences)	02-11-2006	MRP	NL	Article 10 (1)
	Propofol Primex ^d^(Primex Pharmaceuticals)	17-04-2009	MRP	FI	Article 10 (1)
	Propofol Norameda (UAB Norameda)	28-04-2011	DCP	DE	Article 10 (1)
	Propofol BioQ Pharma (BioQ Pharma)	06-07-2012	DCP	NL	Article 10 (1)
	Propofol Sandoz (Sandoz)	06-07-2012	DCP	NL	Article 10 (1)
	Ripol (Corden Pharma)	21-02-2013	DCP	IT	Article 10 (1)
	Propofol MCT/LCT Fresenius pre-filled syringe (Fresenius Kabi)	12-03-2013	DCP	DE	Article 10 (1)
	Propofol Demo (Demo)	03-05-2017	DCP	PT	Article 10 (1)
Clexane^®^ 2000–15,000 IU (Sanofi-Aventis) Enoxaparin sodium					
	Inhixa	15-09-2016	CP	N/A	Article 10 (4)
	Thorinane	15-09-2016	CP	N/A	Article 10 (4)
	Enoxaparin Becat	24-03-2017	DCP	DE	Article 10 (4)
	Enoxaparin Crusia	24-03-2017	DCP	DE	Article 10 (4)
	Ghemaxan	05-04-2018	DCP	United Kingdom	Article 10 (4)

CP, centralised procedure; DCP, decentralised procedure; MRP, mutual recognition procedure; NP, national procedure; MAH, marketing authorization holder; RMS, reference member state; CZ, Czech; DE, Germany; DK, Denmark; ES, Spain; FI, Finland; FR, France; GR, Greece; IT, Italy; MT, Malta; NL, Netherlands; PT, Portugal; SE, Sweden; United Kingdom, United Kingdom.

a This refers to the MAH, listed for the RMS, as in some cases different MAHs exist in different Member States.

b The authors could not retrieve any (publicly) available information on the application procedure.

c Refers to a new dosage form (5 mg/ml) approved *via* a hybrid application procedure.

d This generic application was transferred *via* an informed consent application procedure from Bayer to Primex.

Nanocolloidal solutions of iron carbohydrates for intravenous applications are another example of frequently used nanomedicines. The first nanotechnology-based intravenous iron product was introduced in the 1950s and is now known as Venofer^®^. To overcome the high toxicity of iron (II) salts, iron in the form of polynuclear Fe(III)-oxyhydroxide core stabilized by a carbohydrate shell was developed. Intravenously applied Venofer^®^ nanoparticles have been shown to be tolerated at more than 20-fold higher 50% lethal dose (LD 50) levels, compared to iron sulphate solutions in mice (Geisser et al., 1992).

After administration, the iron carbohydrate particles interact with the innate immune system for uptake and release of bioavailable iron (Geisser and Burckhardt, 2011; Koskenkorva-Frank et al., 2013). It is assumed that the characteristics of the nanoparticles affect the fate and disposition in the body (Toblli et al., 2009a; Toblli et al., 2009b; Toblli et al., 2012; Toblli et al., 2015; Toblli et al., 2017). There is a plethora of evidence showing that iron sucrose follow-on products from different manufacturers have different efficacy and safety profiles despite most of them complying with the USP monograph quality requirements (Rottembourg et al., 2011b; Lee et al., 2013; Agüera et al., 2015). Since the structural and functional relationships are not fully understood and, hence, the clinically meaningful critical quality attributes (CQAs) are not fully identified, the manufacturing process defines the product and is crucial for the consistency and quality of the end product and its clinical performance. A robust manufacturing procedure needs to be in place and thoroughly sustained in order to ensure batch-to-batch consistency. Hence, the call for a harmonised centralised regulatory process to ensure the highest safeguards against patient safety issues.

It should be noted that whilst the centralised procedure is already compulsory in a number of situations^1^, including all those products containing new active substances in, for example, the field of oncology and viral diseases, it does not cover all nanomedicines and nanosimilars. This means that a large number of innovative nanomedicines (including the COVID mRNA) go through the centralised procedure by default. The gap in the system is that for many nanomedicines (i.e., for other indications), it is not yet compulsory for all follow-on/nanosimilars.

As described, for example, by Klein et al., current different routes obtained for marketing approval allows the same nanosimilar to be registered under a variety of brandnames in different countries. This means that when adverse event cases are reported, it is hard to link these patient safety incidences.

As such, nanosimilars would benefit from a mandatory centralised procedure, as this will guarantee consistency in the scientific evaluation of such follow-on products. Another benefit of the centralised procedure is the guarantee of centralised safety monitoring and the obligation for the use of a single brand name throughout the EU. This will facilitate better traceability and adequate identification of product-specific safety issues for nanosimilars.

## Results

In 2020, a comprehensive scientific report (Patient Safety and Nanomedicines, 2020) was produced by the EAASM, and a leading politician who sits on the ENVI Committee, namely, MEP Maria da Graca Carvalho (Official website of MEP Petar Vitanov, 2021) (EPP, Portugal), stated in the foreword that (Patient Safety and Nanomedicines, 2021)

“A strong fit-for-purpose regulatory framework is needed, in order to build on all of the current knowledge and expertise. Only then will we be able to have new treatment opportunities that will benefit patients in a timely and safe way.”

An outreach petition encouraging interested parties to join have resulted in the following organizations (Table 3) becoming part of the Nanomedicines Regulatory Coalition, namely, European Alliance for Access to Safe Medicines, European Cancer Patient Coalition, European Liver Patients’ Association, European Parkinson’s Disease Association, European Renal Association, European Specialist Nurses Organization, International Alliance of Patients’ Organizations.

**TABLE 3:** Nanomedicines Regulatory Coalition.

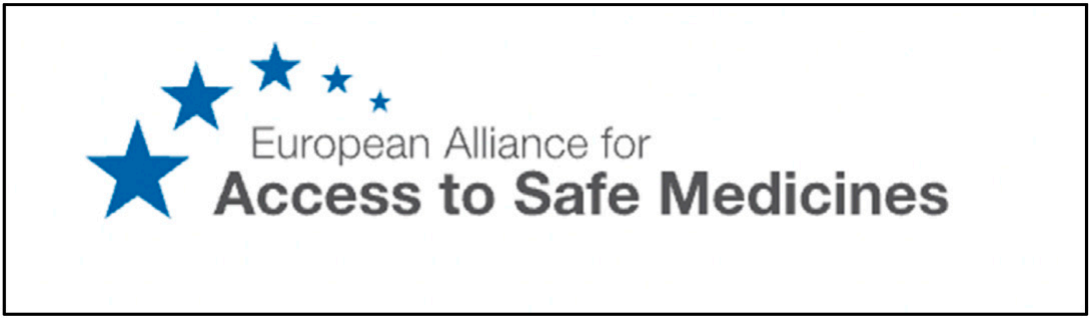	The European Alliance for Access to Safe Medicines (EAASM) is an independent pan-European not-for-profit organization dedicated to protecting patient safety by ensuring access to safe medicines - falsified medicine awareness/safer use of off-label medicines/medication errors/nanomedicine and nanosimilar regulatory clarity

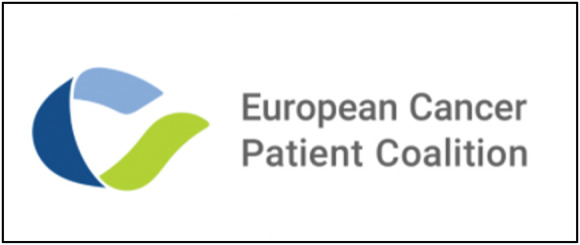	The European Cancer Patient Coalition (ECPC) ensures that the voice of cancer patients in Europe is represented in all relevant policy-making decisions in the European Union. It works to anticipate and formulate legislation taking place at the EU level and actively participates in the EU legislative health policy process

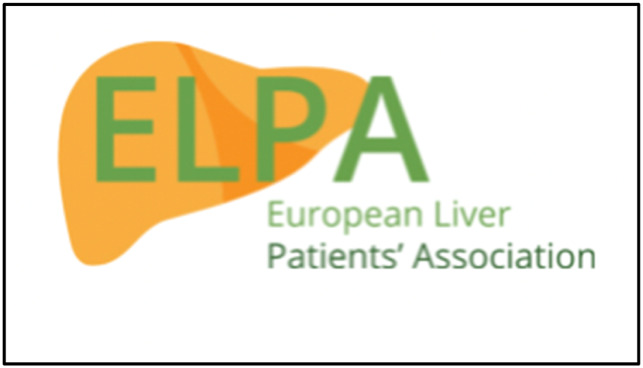	ELPA’s aim is to promote the interests of people with liver disease: to highlight the size of the problem; to promote awareness and prevention; to address the low profile of liver disease as compared to other areas of medicine; to share experience of successful initiatives; to work with professional bodies such as The European Association for the Study of the Liver (EASL) and with the EU, to ensure that treatment and care are harmonised across Europe to the highest standards
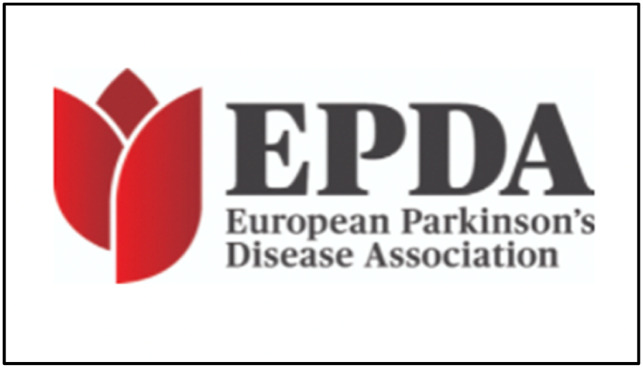	The European Parkinson’s Disease Association (EPDA) has been championing and working with the global Parkinson’s community for nearly 30 years, providing information and resources to all stakeholders whilst raising awareness of the disease’s complexities and impact and advocating for concrete policy change that benefits the Parkinson’s community
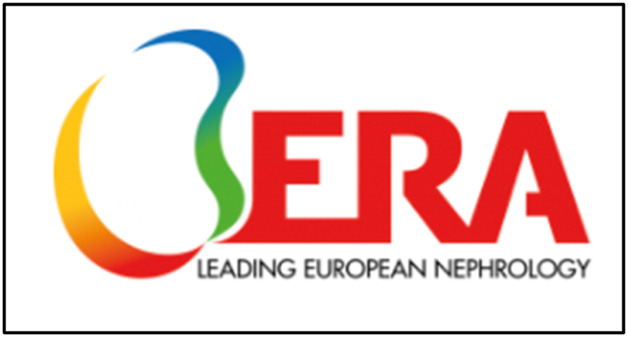	The European Renal Association (ERA) objectives are the advancement of medical science by promoting fundamental and clinical advances in the field of nephrology, dialysis, renal transplantation, hypertension, and related subjects achieved by collaboration, education, research, raising public awareness, and career opportunities whilst enhancing professional networking
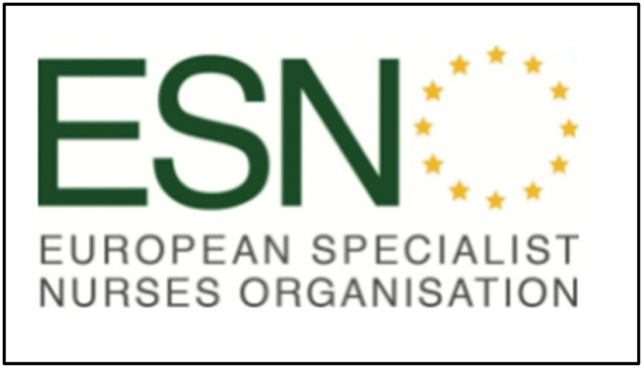	ESNO promotes and represents the interests of specialist nurses in Europe. Through collaboration with stakeholder organizations and utilizing advanced science, it pursues recognition under the Directive of Professional Qualification. ESNO contributes to effective co-operation between health professionals, organizations, institutes, and agencies, thereby setting professional standards for education and continuing development

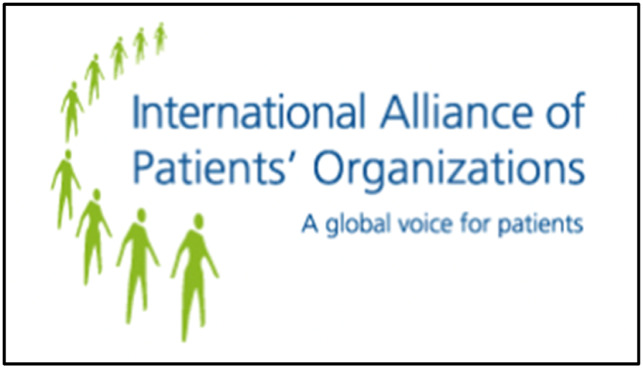	The International Alliance of Patients’ Organizations (IAPO) is a unique global alliance representing patients of all nations across all disease areas. It has 300 member organizations from 71 countries representing 50 disease areas. IAPO’s vision is to see patients placed at the center of healthcare and its mission is to help build patient-centered healthcare worldwide

In addition, the EAASM has held focused interviews with leading MEPs (MEP Cyrus Engerer, 2021) whose major interest is health related and who were co-signatories to a letter sent to the EU Commissioner for Health and Food Safety, Ms. Stella Kyriakides, on 30 June 2021, to which a positive reply was received, and the Commissioner’s statement can be seen in Table 4 ^2^.

**TABLE 4:** Letter from the EU Commissioner for Health.

“You rightly mention that a key objective of the pharmaceutical strategy for Europe is to enable innovation and adapt the European medicines regulatory framework to cutting-edge products and scientific developments
With this objective in mind, we will revise the pharmaceuticals legislation. We have already published a Roadmap/inception impact assessment, which outlines the main policy considerations to adapt the current system of authorisations and the possibility to change the scope of the centralised application procedure for innovative products. While I am not able at this moment to prejudge the result of this analysis, let me reassure you that the final policy directions will be based on a thorough impact assessment and extensive public consultations.”

The lead rapporteur on the European Parliament INI report, MEP Dolors Montserrat^3^ (EPP, Spain), charged with challenging the Commission’s legislative proposal, was receptive to the recommendations that nanomedicines should be specifically mentioned in the INI report. The amendments will be voted in the EU Parliament plenary session in Q4 2021, and there is confidence that the inclusion of nanomedicines and nanosimilars will remain and thus be transposed into the EU Pharmaceutical Strategy master policy document that will ultimately result in new legislation.

For the current amendments that are in the Environment, Public Health, and Food Safety (ENVI) Committees’ INI report^4^ 2021/2013/INI 08/11/2021, see Table 5.

**TABLE 5:** ENVI report -2021/2013/INI.

25. Calls on the Commission to build on the work of Europe’s Beating Cancer Plan and ensure that Europe becomes the worldwide centre of excellence for R&D in emerging, innovative fields of medicine; underlines that state-of-the art technologies, such as nanomedicines, stand to provide solutions to current treatment challenges in areas such as cancer and cardiovascular diseases; highlights that these innovative fields of medicine should be authorised by the centralised approval framework for nanomedicines
101. Urges the Commission and the EMA to consider the full lifecycle of all innovative medicines and therapies, including gene and cell therapies, personalised medicine, nanotechnology and next-generation vaccines, and ensure a fit-for-purpose framework for off-patent competition at the time of loss of exclusivity; calls on the Commission to establish a regulatory framework for nanomedicines and nanosimilar medicines, and calls for these products to be approved through a compulsory centralised procedure

MEP Petar Vitanov (Official website, 2021) (S&D, Bulgaria) was interviewed by the Parliament Magazine (Parliament Magazine Nanomedicines and Nanosimilars, 2021) and clearly stated the following:

“As an MEP actively involved in health care, and with the progress of the Pharmaceutical Strategy for Europe, it is the right time to set the scene for building a pan-European medical agency consensus so that regulatory weaknesses can be addressed through a robust regulatory pathway and thus provide medicines with the highest quality, safety and efficacy profiles for European patients.”

Following on from two Parliament round-table events, a third follow-up event is tabled for Q3 2021.

## Conclusion

In the comprehensive Master Research protocol (Van Trier, 2021) thesis entitled “European stakeholders’ perspectives on the therapeutic opportunities and the regulatory challenges associated with nanomedicines,” the main conclusions under Section 6.3, “*The Future of Nanomedicines*,” were as follows (see Table 6):

**TABLE 6:** “European stakeholders’ perspectives on the therapeutic opportunities and the regulatory challenges associated with nanomedicines.” Section 6: “The Future of Nanomedicines.”

“All interviewees saw a fairly bright future for nanomedicines. The number of MA applications is steadily increasing and the topic is more and more discussed at large conferences. Partly due to the accomplishments with the COVID-19 vaccines, it was expected that fundamental research into the size-specific properties of nanodrugs will receive a further boost and the use of already successful technologies such as encapsulation in liposomes will be extended to new indications. The question remains whether nanodrugs will mainly continue to be delivery vehicles or whether a transition to new stand-alone substances will be made. The latter would further stimulate the commercial potential of nanomedicines. In addition, it was expected that the importance of follow-on products will continue to increase in the search for more affordable medicines for a wide audience
However, additional clarification of the regulatory landscape will be necessary to fully realize the potential of these drugs. Regulatory authorities must be ambitious and continue to set themselves the goal of optimizing the regulation of innovative medicines and translating an increase in knowledge into improved guidelines. What has been learned from the situation with biologicals is that this evolution is slow. The will to change European pharmaceutical legislation and include nanomedicines as a distinct concept into the legal framework is rather small. As a result, changes such as a mandatory central procedure or a specific pathway for nanomedicines’ follow-on products may not be quickly realized after all.”

This last sentence is a significant cause for concern and so the Nanomedicines Regulatory Coalition under the umbrella of the EAASM intends to continue its strong advocacy program to ensure the following:

• all nanomedicines and nanosimilars be assessed by the EMA Centralised Regulatory Procedure.
• a harmonization of information requirements of regulators in order to correctly characterize nanomedicines
• production of a scientific consensus on definitions for nanomedicines across Europe
• improved education and a fostering of awareness on the complexity and sophistication of nanomedicines among policymakers, prescribers, payers, and patients. This is especially relevant when it centers on issues of interchangeability

## Footnotes

1
https://www.ema.europa.eu/en/about-us/what-we-do/authorisation-medicines#scope-of-the-centralised-procedure-section

2
https://ec.europa.eu/health/human-use/strategy_en

3
https://www.europarl.europa.eu/meps/en/197711/DOLORS_MONTSERRAT/home

4
https://www.europarl.europa.eu/doceo/document/A-9-2021-0317_EN.pdf

## Abbreviations

ANDA, Abbreviated New Drug Application; CDER, Center For Drug Evaluation And Research; CQA, critical quality attributes; EAASM, European Alliance for Access to Safe Medicines; EMA, European Medicines Agency; ENVI Committee, the Committee on the Environment, Public Health and Food Safety European Parliament; INI report, own-initiative report of the European Parliament; IND, investigational new drug application; LD, lethal dose; MEP, Member of the European Parliament; NBCD, non-biological complex drugs; NDA, new drug application; USP, United States Pharmacopoeia.
